# Homicides during the Barranquilla Carnival, Colombia: A 10 Year Time-Series Analysis

**DOI:** 10.3390/ijerph17010035

**Published:** 2019-12-19

**Authors:** Jhon Albert Guarin-Ardila, Rossycela Montero-Ariza, Claudia Iveth Astudillo-García, Julián Alfredo Fernández-Niño

**Affiliations:** 1 Facultad de Medicina, Fundación Universidad del Norte, Barranquilla 081007, Colombia; ajguarin@uninorte.edu.co (J.A.G.-A.); rossycelam@uninorte.edu.co (R.M.-A.); 2 Servicios de Atención Psiquiátrica, Secretaría de Salud, Ciudad de Mexico 11410, Mexico; 3 Departamento de Salud Pública, Universidad del Norte, Barranquilla 081007, Colombia; aninoj@uninorte.edu.co

**Keywords:** homicide, violence, time-series studies, Colombia

## Abstract

Homicides are currently the third leading cause of death among young adults, and an increase has been reported during holidays. The aim of the present study was to explore whether an association exists between Carnival in Barranquilla, Colombia, and an increase in homicides in the city. We used mortality records to identify the number of daily homicides of men and women throughout the week of Carnival, and we compared those with records from all of standard days between 1 January 2005 and 31 December 2015. Conditional fixed-effects models were used, stratified by time and adjusted by weather variables. The average number of homicides on Carnival days was found to be higher than on a standard day, with an OR of 2.34 (CI 95%: 1.19–4.58) for the occurrence of at least one male homicide per day during Carnival, and 1.22 (CI 95%: 1.22–7.36) for female homicides, adjusted by weather variables. The occurrence of homicides during Carnival was observed and was similar to findings for other holidays. Given that violence is a multifactorial phenomenon, the identification of the factors involved serves as a basis for evaluating whether current strategies have a positive effect on controlling it.

## 1. Introduction

According to data from the Global Health Observatory (GHO), 457,000 homicides occurred worldwide in 2012 (a rate of 6.7 per 100,000 inhabitants), constituting the third leading cause of death for the population between 15 and 44 years of age [1]. In Colombia, violence is among the most important public health concerns. For that same year, homicide explained 8.85% of disability-adjusted years lost for all age groups and 18.07% for people between 15 and 49 years of age [2], with a homicide rate of 43.9 per 100,000 inhabitants for the latter age group [1].

Historically, a temporal behavior in the occurrence of homicides has been reported [3], which could be related to variations in weather (temperature, humidity, and rainfall) [4], among other factors. Different theories have arisen to explain this association [5]. For example, Quetelet proposed what is known as the temperature–aggression hypothesis, which suggests that high temperatures increase discomfort, which increases aggression [6]. There is also the general aggression model (GAM), which proposes that weather variables, especially temperature, can lead to aggressive thoughts and even violence, although it presumes that there is a temperature limit above which these behaviors decrease [7,8]. Nevertheless, the lack of solid evidence for an association between temperature and homicides provides a basis for considering that homicide seasonality may be related not only to the physical environment, but might also have a social component [3]. This explanation would support the fact that an increase in homicide rates has been reported on and around special dates, such as events or holidays such as Christmas, New Year, and Thanksgiving [4,6,7,8,9,10].

The “routine activity theory” [11] is one possible explanation for the relationship between special dates and homicides. This suggests that people tend to have more social interactions along with more recreational activities on those dates, thereby creating more spaces for socializing where violent conflicts that may involve criminal behavior could be generated, which could be reinforced by the presence of other risk conditions such as consumption of more alcoholic beverages [6]. In addition, these events could result in more fatalities, given that the availability and quality of medical care on these days tends to decrease, since many health professionals do not work on holidays [9].

In Colombia, the Barranquilla Carnival is one of the most important cultural festivals in the country. Each year, folklore, dance, and music are celebrated, bringing out the particular joy of the Caribbean and sharing it with visitors. In 2003, UNESCO declared the festival a Masterpiece of the Oral and Intangible Heritage of Humanity [12]. Carnival begins on the Friday before Ash Wednesday, which is the first day of Lent on the Catholic Liturgical Calendar. It last for at least five days, during which many activities take place in which the participants indulge in parties, collective enjoyment, and disinhibition, along with increased alcohol consumption and the risk of aggression [13,14].

Barranquilla has seen a representative decrease in the homicide rate over recent years, dropping to 26.4 homicides per 100,000 inhabitants in 2013. Since then, a relative increase has been observed, with 2015 having the second highest rate in recent years, with 34.6 homicides per 100,000 people, 93% of whom were male [15]. Given this scenario, lines of action to prevent violence need to be developed based on knowledge that is specific to the particular context of a region. This is especially needed in Latin America, considering that few studies have investigated the association between homicide and holidays [6,10], and fewer still have explored its relationship with activities that are as representative as Carnival.

By analyzing homicide patterns on holidays, it is possible to prevent actions by implementing strategies that are specific to a particular space and time. It is important to evaluate the associated factors, and to promote and evaluate interventions, especially in Latin America where violence is an important public health concern.

To our knowledge, no studies have estimated changes in the number of homicides on and around the time of the Barranquilla Carnival or other holidays. Therefore, the present study aimed to estimate the adjusted changes in the number of homicides during Carnival and compare those to a standard day for the period 2005 to 2015, for men and women and adjusted by weather variables.

## 2. Materials and Methods

### 2.1. Study Area

Barranquilla is one of the most important cities in Colombia. It is located on the western banks of the Magdalena River, near its mouth at the Caribbean Sea. It is home to 1,223,616 people and is a center of economic growth. The main economic activities in this city include industrial manufacturing and metal mechanics, and production of food and beverages, textiles, and chemical substances, making it the third largest air, sea, river, and communications port in Colombia [16].

### 2.2. Study Design

A multi-temporal, ecological study was carried out in a single city. The unit of observation was the city/day for the period between 1 January 2005 and 31 December 2015, using daily homicide counts for the City of Barranquilla differentiated by gender.

### 2.3. Variables and Sources of Data

#### 2.3.1. Response Variable

The response variable was homicide count. The number of homicides per day was obtained based on codes CIE-10 Y089 and Y099 for the main cause of death. These counts were obtained from mortality databases provided by the National Statistics Department (DANE, Spanish acronym), for the study period. The exposure variable for the analysis was the total population of the city, which was obtained based on the projection of the total population for each year according to the population projections by DANE. Given the large number of zeros, a dichotomous variable was generated for all days (1 = at least one homicide, 0 = none).

#### 2.3.2. Independent Variables

The independent variable was Carnival. The time at which Carnival is held is determined by the Catholic calendar. It officially begins on the Saturday before Ash Wednesday, which varies from year to year according to the lunar calendar. It is divided into two stages, pre-Carnival (preparatory celebrations) and Carnival, the latter being the objective of this study. Carnival lasts for four days, each one having a main celebration: the Battle of Flowers on Saturday, the Great Tradition and Folklore Parade on Sunday, the Great Fantasy Parade and Orchestra Festival on Monday, and lastly, special story dance festivals, litanies, and the Death of Joselito on Tuesday [17]. Since the dates vary each year in Colombia, official historical calendars were used to manually recover this information.

An indicator variable was generated for the analysis, in which 1 indicated being at Carnival and 0 indicated not attending. The day of the week was used as a reference for both cases, such that a day during Carnival was compared to the same day of a standard week. Carnival was held from Saturday through Tuesday; therefore, Wednesday, Thursday, and Friday were considered post-Carnival days.

#### 2.3.3. Covariables

Weather variables were the covariables in this study. The weather data that were obtained included temperature (°C), relative humidity (%), and rainfall (mm). These were taken from the weather stations at the Barranquilla airport, which records information throughout the year.

Other holidays were also covariables in this study. The following holidays were also included: Christmas (24 and 25 December), New Year (31 December and the first day of the following year, 1 January), Mother’s Day (second Sunday of May every year), and Colombian Independence Day (celebrated on 20 July).

### 2.4. Statistical Analysis

The mean homicides for men and women were estimated for each holiday, along with their respective measures of dispersion (mean and standard deviation). The percentage of days with at least one homicide was also calculated, along with the respective 95% confidence interval. Conditional fixed-effects models were adjusted by the day of the week and month of the year. Time-stratified conditional fixed-effects models (grouped by day of the week and month) were estimated in order to calculate odds ratios for the occurrence of at least one homicide per day, by sex, with a standard day as reference. In addition, a binomial fixed-effects regression model for total daily homicides per sex was adjusted. Both models were stratified by time in order to reflect the structure of the correlation of the observations when generated on the same day of the week and month of the year [18]. Weather variables were used as adjustment variables and goodness-of-fit was verified for both models. An association was considered to be statistically significant when *p* < 0.05.

Lastly, an estimate was obtained of predicted cases during Carnival versus expected cases on the same day of a standard week. All the analyses were performed with Stata v.14 [19].

## 3. Results

Table 1 presents the distribution of the occurrence of homicides in Barranquilla on the holidays studied, from 2005 to 2015. Taking a standard day as reference, we can see that for this city during the study period, the average homicide count for men on a standard day was 0.82, with a standard deviation (SD) of 0.96 homicides/day. In comparison, the days with higher averages were Carnival (mean: 1.39, SD: 1.38), Christmas (mean: 1.36, SD: 1.40), and Mother’s Day (mean: 1.90, SD: 1.66). Given the high number of zeroes, the proportion of days that had at least one homicide was used for the analysis. As a result, Mother’s Day was the holiday with the largest proportion of days with at least one homicide (80%), followed by Carnival/post-Carnival and Christmas (72.73% each). For the other holidays studied, the occurrence of one homicide was similar to a standard day.

With regard to sex, the number of homicides was much lower for women than for men, and the daily averages were also much lower for women. Nevertheless, the homicide average for women increased during New Year (mean: 0.18, SD: 0.39) and Carnival (mean: 0.14, SD: 0.35). For women, the proportions of days with at least one homicide were also New Year 18.18%, Carnival 13.64%, and Mother’s Day 10% (Table 1).

Table 2 presents the results from the logistic regression model for the occurrence of at least one homicide/day for men and women in Barranquilla. For men, a positive association was found for Carnival days as compared with standard days, with an odds ratio (OR) of 2.34 (CI95%: 1.19–4.58). A positive association was also found with Christmas, although this was only marginally significant (OR = 2.27; CI 95%: 0.89–5.82). No statistically significant association was found for New Year, Mother’s Day, or Independence Day. For women, a statistically significant relationship was also found for Carnival (OR = 3.00; CI 95%: 1.22–7.36) and for New Year (OR = 4.47; CI 95%: 1.46–13.72), and no statistically significant association was found for Christmas or Mother’s Day. In addition, given it is conditioned by time, the variances of the homicides tended toward zero; therefore, it was not possible to compare the effect of Independence Day with standard days.

Table 3 shows the binomial fixed-effects regression models for total daily homicides for men and women. As can be seen, for men, the number of homicides increased during Carnival, with a rate ratio (RR) of 1.66 (CI 95%: 1.23–2.24). The RR was 1.64 (CI 95%: 1.07–2.49) for Christmas and 2.26 for Mother’s Day (CI 95%: 1.28–4.02). No statistically significant association was found for New Year or Independence Day. For women, the expected number of homicides increased during Carnival, with a rate ratio (RR) of 2.54 (CI 95%: 1.04–6.18), and New Year (RR = 3.58; CI 95%:10.19–10.74).

**Figure 1:**
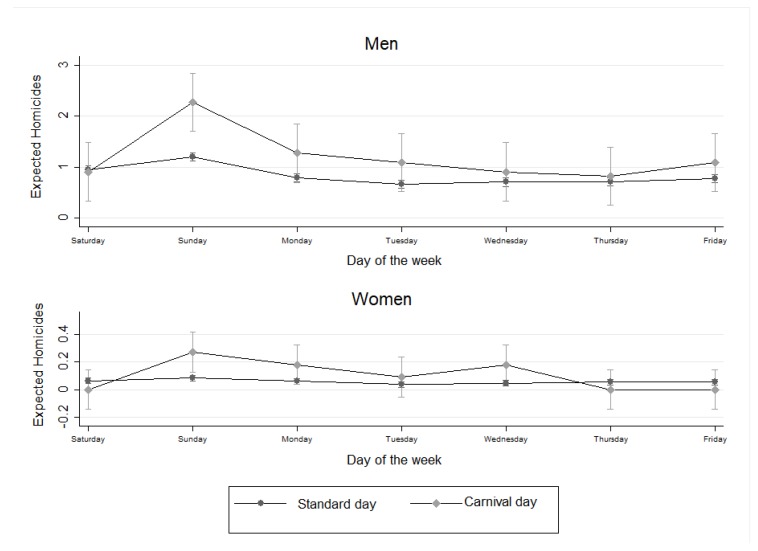
Expected homicides for men and women on a standard day versus Carnival/post-Carnival days, Barranquilla, 2018.

Figure 1 shows the differences between expected homicides for men and women on a day during Carnival/post-Carnival versus expected homicides on the same day of a standard week. As can be seen, the differences were more notable and were statistically significant on the Sunday of Carnival, and then decreased between Monday and Thursday (Wednesday, Thursday, and Friday being post-Carnival days). This indicates that the difference in the number of homicides was particularly important on the Sunday of Carnival.

## 4. Discussion

An increase in homicides was observed on Carnival days compared to standard days. A similar increase was also observed on other major holidays, such as Christmas and New Year. It is important to note that while this was roughly double in relative terms, in absolute values it represented an increase of one to two homicides per day. Therefore, the “effect” had a mild population impact, and thus it should be noted that the Barranquilla Carnival is a relatively peaceful celebration as compared to other holidays in the same city and in other countries.

This increase in homicide numbers during holidays (controlled by weather variables) is consistent with the routine activity theory [11], which suggests that certain external events affect the activity patterns and habits of an individual, and that these changes could create situations of conflict that would not occur if the people were involved in structured environments such as school or work [20]. In this way, major holidays that involve changes in activity patterns and mass gatherings, combined with alcohol consumption, could contribute to an increase in situations that lead to conflict and violent behavior, as compared to minor holidays during which daily activities do not change [7,20].

A change in routine activities could help to explain an increase in homicides during Carnival for both men and women, as well as the association between homicides and other holidays such as Christmas and New Year, which has previously been reported [21]. Other crimes committed on those days have also been reported [20], and these holidays have therefore been considered a risk factor for overall mortality [9], and particularly for deaths from cardiac causes [22,23]. Nevertheless, Mother’s Day has only been associated with suicides in Mexico [24]. In the case of Barranquilla, this increase in homicides seems to exist but is relatively minor, which suggests that this effect is mitigated by the nature of the celebration itself, as well as by cultural particularities and the city’s security policies.

Another important situation that occurs during major holidays or mass events is the gathering in one environment of diverse people with different backgrounds, where the cultural differences that they may have could induce aggressive behavior, especially if the crowd is diverse (as occurs in places such as bars, or national or local celebrations when people come from different places). Thus, some people will likely begin to exhibit asocial behavior that, combined with overcrowding and excessive alcohol consumption, lead to frustration and irritation, which could cause aggression and a consequent increase in violence and homicide indices [25]. In the case of the Barranquilla Carnival, while there is an influx of people from outside the city, they represent less than 5% of the deaths.

With regard to the days of the week on which more homicides have been observed, reports have consistently shown that homicides increase on weekends (Friday to Sunday) as compared with weekdays [6,26,27]. This is particularly notable when compared to Carnival/post-Carnival days, when a significant increase was found on Sunday, and even suggests the possibility that homicides recorded on Monday could be explained by a delay in the records, that is, some homicides may be related to activities that began on Sunday night, but the crime itself or the time it was recorded to have occurred after midnight, on Monday, the following day [6].

A homicide is an event that results from a combination of various factors interacting in the same space and at the same time [4]. Aggressive behavior can arise from a reciprocal interaction between the person and the environment [25]. Consistent evidence about the relationship between holidays and homicides could serve as an important guide for designing and implementing prevention strategies. Nonetheless, given that homicide is a multifactorial event, more investigations need to be conducted about the factors that are involved.

Risk factors that lead to violent behavior in specific places need to be determined. For example, the organizational practices and physical characteristics of establishments that serve alcohol influence the occurrence of violent behavior [28]. In their review of risk factors in bars, Green and Plant [29] determined that these can be divided into internal and external. Internal factors include the physical characteristics and atmosphere of the bars (for example, layout, crowding), organizational factors (for example, drink promotions, types of entertainment offered), the customers’ characteristics (for example, sex and age), and the choice of beverage. External factors include variables such as location and density.

Likewise, the geolocation of homicides can be considered [30], and patterns in other cities that hold Carnivals can be compared, as can the spatial and temporal variations of other types of illicit activity (robbery, rape). The patterns of the perpetrators’ characteristics can also be reconstructed, as well as the profiles of the victims, considering that studies have shown that the relationship between violence and geographic location can be complemented by a social inequality perspective [31,32].

Identifying the factors that are related to violence and homicides and inserting them into a framework such as routine activity theory could be useful not only for explaining the periodicity of crime, but also for training security personnel for specific places, events, and days [33]. This type of study could also be used to review and evaluate regulatory policies for the sale and consumption of alcohol, and to increase surveillance of other psychoactive substances, especially on days that are identified as having a higher risk, such as weekends, holidays, and the Sunday of Carnival. A positive impact on decreasing violence during holidays could be achieved by implementing multilevel interventions that take into account the structural, environmental, and individual factors that are associated with violence, based on the triangulation of theoretical perspectives that help to explain the interaction between violence and the social and physical context in which it occurs [25]. New tools such as crowd studies could also contribute to this aim [34].

In 2017, the mayor of Barranquilla issued Ordinance No. 0192 to establish policies for gatherings, thereby developing regulations for the sale and consumption of alcohol as well as time limits for public gatherings [35]. A security plan for Carnival was established in 2018, which provides a specific number of police officers based on the size and seating capacity of each event [36]. Nevertheless, international recommendations for decreasing violence indicate the need to analyze the costs and benefits of these and other prevention measures in order to generate evidence, especially so that low- and middle-income countries can adopt measures based on evidence related to the particular circumstances of an environment [37].

This study had some limitations that should be taken into account. For example, the design had the typical limitations of ecological approaches, and the estimates and conclusions can only be interpreted at the population level, since the study did not take into account individual differences. For instance, in this analysis, it was not possible to determine the residence of the deceased person, which certainly constitutes another limitation of this study. In this way, it is not possible to establish whether we were observing an effect on the seasonal behavior of the series of homicides in the municipality, or associated with phenomena involving external visitors. In addition, if the behavior of homicides were entirely explained by routine activity theory, then spatial variations would be expected on the various days of the week, as well as during different periods of the day [6]. Nevertheless, that information was not available for the analysis herein.

With regard to the quality of the data, Colombia’s official mortality records from DANE were verified and validated by experts in the death coding system, and it was estimated that these records represent approximately 95% of the deaths in urban areas. Cendales and Pardo [38] evaluated the quality of the information on death certificates between 2002 and 2006 and found that 92.8% of the deaths were correctly certified and coded, thereby concluding that the quality of Colombia’s National Mortality Registry is adequate.

Violence is a multifactorial phenomenon. The extent to which available data are used to understand the magnitude and nature of the problem, to identify its causes, to formulate strategies, and evaluate the effects of those measures, we will have the tools needed to contain it. Holidays and other events such as Carnival are social activities that enable both the expression of joy and displays of culture, which do not necessarily have to be linked to criminal activities.

## 5. Conclusions

Homicides do increase during Carnival, but similarly to other festivities, which is consistent with routine activity theory, which suggests that major holidays are more likely to affect and alter the normal daily activities of people converging from several places in a setting that frequently involves the consumption of alcohol and other substances. Being a festivity that characterizes Barranquilla, Colombia, the efforts made by government authorities to control violence during festivities in general should be evaluated.

## Figures and Tables

**Table 1:** Distribution of total daily homicides by holiday, Barranquilla, 2005–2015.

	Men		Women	
Holiday	Mean (SD)	% of Days with at Least One Homicide (CI 95%)	Mean (SD)	% of Days with at Least One Homicide (CI 95%)
Standard day (*n* = 3908)	0.82 (0.96)	54.04 (52.48–55.61)	0.06 (0.25)	5.25 (4.55–5.94)
Carnival/post-Carnival (*n* = 44)	1.39 (1.38)	72.73 (59.41–86.04)	0.14 (0.35)	13.64 (3.68–23.90)
Christmas (*n* = 22)	1.36 (1.40)	72.73 (53.67–91.78)	0.09 (0.29)	9.09 (0–21.39)
New Year (*n* = 22)	0.91 (1.48)	40.91 (19.87–61.94)	0.18 (0.39)	18.18 (1.68–34.68)
Mother’s Day (*n* = 11)	1.90 (1.66)	80.00 (53.86–100.00)	0.10 (0.32)	10.00 (0–29.6)
Independence Day (*n* = 11)	1.00 (1.18)	54.55 (23.67–85.42)	0.00 (0.00)	0 (NE)
Total	0.84 (0.98)	54.34 (52.80–55.88)	0.06 (0.25)	5.43 (4.72–61.28)

SD: standard deviation, CI: confidence interval, NE: not estimable.

**Table 2:** Fixed-effects logistic regression model for the occurrence of at least one homicide/day for men and women in Barranquilla, 2005–2015.

	Men				Women			
Reference (Standard Day)	Odds Ratio	CI 95%		*p* > z	Odds Ratio	CI 95%		*p* > z
Carnival/	2.34	1.19	4.58	0.01	3.00	1.22	7.36	0.02
Christmas	2.27	0.89	5.82	0.09	1.88	0.43	8.14	0.40
New Year	0.70	0.29	1.69	0.42	4.47	1.46	13.72	0.01
Mother’s Day	2.85	0.59	13.76	0.19	2.21	0.27	17.85	0.46
Independence Day	1.04	0.28	3.87	0.96	Not estimable			

**Table 3:** Binomial fixed-effects regression model for total daily homicides for men and women, Barranquilla, 2005–2015.

	Men				Women			
Reference (Standard Day)	Rate Ratio	CI 95%		*p* > z	Rate Ratio	CI 95%		*p* > z
Carnival	1.66	1.23	2.24	0.00	2.54	1.04	6.18	0.04
Christmas	1.64	1.07	2.49	0.02	1.68	0.39	7.25	0.48
New Year	1.19	0.62	2.28	0.61	3.58	1.19	10.74	0.02
Mother’s Day	2.26	1.28	4.02	0.01	1.97	0.24	15.76	0.52
Independence Day	1.34	0.66	2.72	0.41	Not estimable			
